# Test–retest stability of spontaneous brain activity and functional connectivity in the core resting‐state networks assessed with ultrahigh field 7‐Tesla resting‐state functional magnetic resonance imaging

**DOI:** 10.1002/hbm.25771

**Published:** 2022-01-19

**Authors:** Hasan Sbaihat, Ravichandran Rajkumar, Shukti Ramkiran, Abed Al‐Nasser Assi, Jörg Felder, Nadim Jon Shah, Tanja Veselinović, Irene Neuner

**Affiliations:** ^1^ Institute of Neuroscience and Medicine, INM‐4 Jülich Germany; ^2^ Department of Medical Imaging Arab‐American University Palestine (AAUP) Jenin Palestine; ^3^ Department of Psychiatry, Psychotherapy, and Psychosomatics RWTH Aachen University Aachen Germany; ^4^ JARA—BRAIN—Translational Medicine Aachen Germany; ^5^ Department of Neurology RWTH Aachen University Aachen Germany; ^6^ Institute of Neuroscience and Medicine, INM‐11, Forschungszentrum Jülich GmbH Jülich Germany

**Keywords:** 7 Tesla, functional connectivity, functional magnetic resonance imaging, high resolution, resting state, test–retest stability, triple‐network model

## Abstract

The growing demand for precise and reliable biomarkers in psychiatry is fueling research interest in the hope that identifying quantifiable indicators will improve diagnoses and treatment planning across a range of mental health conditions. The individual properties of brain networks at rest have been highlighted as a possible source for such biomarkers, with the added advantage that they are relatively straightforward to obtain. However, an important prerequisite for their consideration is their reproducibility. While the reliability of resting‐state (RS) measurements has often been studied at standard field strengths, they have rarely been investigated using ultrahigh‐field (UHF) magnetic resonance imaging (MRI) systems. We investigated the intersession stability of four functional MRI RS parameters—amplitude of low‐frequency fluctuations (ALFF) and fractional ALFF (fALFF; representing the spontaneous brain activity), regional homogeneity (ReHo; measure of local connectivity), and degree centrality (DC; measure of long‐range connectivity)—in three RS networks, previously shown to play an important role in several psychiatric diseases—the default mode network (DMN), the central executive network (CEN), and the salience network (SN). Our investigation at individual subject space revealed a strong stability for ALFF, ReHo, and DC in all three networks, and a moderate level of stability in fALFF. Furthermore, the internetwork connectivity between each network pair was strongly stable between CEN/SN and moderately stable between DMN/SN and DMN/SN. The high degree of reliability and reproducibility in capturing the properties of the three major RS networks by means of UHF‐MRI points to its applicability as a potentially useful tool in the search for disease‐relevant biomarkers.

## Graphical abstract

The growing demand for precise and reliable biomarkers in psychiatry is fueling research interest in the hope that identifying quantifiable indicators will improve diagnoses and treatment planning across a range of mental health conditions. In this work, we investigated the intersession stability of four functional magnetic resonance imaging (fMRI) resting‐state (RS) parameters—amplitude of low‐frequency fluctuations (ALFF) and fractional ALFF (fALFF, representing the spontaneous brain activity), regional homogeneity (ReHo; measure of local connectivity), and degree centrality (DC; measure of long‐range connectivity)—in three RS networks, previously shown to play an important role in several psychiatric diseases—the default mode network (DMN), the central executive network (CEN), and the salience network (SN). Our investigation at individual subject space revealed a strong stability for ALFF, ReHo, and DC in all three networks, and a moderate level of stability in fALFF. Furthermore, the internetwork connectivity between each network pair was strongly stable between CEN/SN and moderately stable between DMN/SN and DMN/SN.

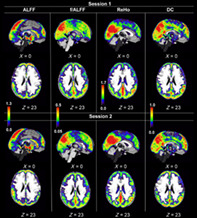

## 1 INTRODUCTION

The increasing demand for precision psychiatry during the last decades (Insel & Cuthbert, 2015) has led to an awareness of the need to establish reliable biomarkers for mental health and different psychiatric conditions. According to the National Institutes of Health Biomarkers Definitions Working Group, biomarkers are “a characteristic that is objectively measured and evaluated as an indicator of normal biological processes, pathogenic processes, or pharmacologic responses to a therapeutic intervention.” (Biomarkers Definitions Working Group, 2001). Consequently, valid biomarkers are increasingly seen as essential for both the precise diagnosis of complex mental disorders and for reliable therapy monitoring.

Since the discovery of temporal correlations obtained by blood‐oxygen‐level‐dependent (BOLD) signal fluctuations during rest in the 1990s (Biswal, Zerrin, Haughton, & Hyde, 1995), the resting‐state (RS) research approach has been increasingly used to map regional interactions in the brain and has considered to have a promising potential in the search for biomarkers for brain‐related disorders (Blautzik et al., 2013; Washington et al., 2013). Indeed, numerous investigations confirm that the human brain is organized into dynamic, intrinsic, resting‐state functional networks (rsNW; Cabral, Kringelbach, & Deco, 2017; Fox et al., 2005; Smith et al., 2013), and the link between serious mental illnesses and abnormal brain connectivity is gaining widespread acceptance (Woodward & Cascio, 2015). Thus, features and patterns derived from spontaneous brain activity and functional connectivity (FC) could be considered as potential neurophysiological biomarkers for various psychopathological phenomena across neuropsychiatric disorders (Blatow, Nennig, Durst, Sartor, & Stippich, 2007; Imperatori et al., 2020).

Resting‐state functional MRI (rs‐fMRI) measures functional connections in the brain via the temporal correlation of low‐frequency (0.01 < *f* < 0.1 Hz) fluctuations in the BOLD fMRI signal. These fluctuations reflect synchronized variations in spontaneous neuronal firing and unconstrained mental activity (e.g., mind wandering; Biswal et al., 1995; Fox & Raichle, 2007; Mason et al., 2007). The main advantage of measuring FC using MRI is its noninvasive nature. Furthermore, the participant is not required to complete an instructed task, meaning that the results are not influenced by the task demands or the efforts and motivation of the participants. Consequently, FC examinations can be considered to be objective. Furthermore, rs‐fMRI examinations are well tolerated by most subjects including patients with severe symptomatology and require a reasonably short acquisition time. Thereby, some authors state that acquisition times of about 6 min have provided adequate sampling to obtain robust results (Van Dijk et al., 2010), whereas others recommend, when possible, longer acquisitions (about 12 min; Hacker, Roland, Kim, Shimony, & Leuthardt, 2019). However, despite the broad applicability, it has to be kept in mind that rs‐fMRI measurements may be hampered by minimal head motions as well as by several physiological effects (e.g., respiration and cardiac pulsatility) and various imperfections in MRI system hardware (e.g., heating of the imaging gradients during experiments; Maknojia, Churchill, Schweizer, & Graham, 2019), thus the preprocessing requires a high degree of diligence.

So far, the transition toward the use of RS connectivity patterns as a biomarker in clinical practice has not yet occurred. Among other things, this requires a better understanding of the microscale brain organization. The development of ultrahigh‐field (UHF) neuroimaging technologies, that is, UHF‐MRI, offer the potential to bridge this shortcoming (Bazin et al., 2014; Dinse et al., 2013; Geyer, Weiss, Reimann, Lohmann, & Turner, 2011). One important consideration hereby is the recognition of the good tolerability of the most UHF‐MRI systems (Theysohn et al., 2007).

The usage of UHF‐MRI entails several advantages. The benefits include increased spatial sampling in the native image, and thus a high spatial resolution (which decreases partial volume effects; Newton, Rogers, Gore, & Morgan, 2012) improved signal‐to‐noise ratio (Triantafyllou et al., 2005), increased sensitivity (Kraff, Fischer, Nagel, Mönninghoff, & Ladd, 2014), enhanced amplitude, and percent of signal change in BOLD signal (Sladky et al., 2013; van der Zwaag et al., 2009), significantly accentuated microvasculature contributions (Duong et al., 2003), and significantly reduced nonspecific mapping signals from large vessels, which together can lead to a deeper understanding of the intrinsic properties of functional brain networks (De Martino et al., 2011; Gorgolewski et al., 2015; Holiga et al., 2018). Moreover, these factors considerably increase the quantity of data obtained per scan and enable the consideration of the individual examination.

However, besides the clear advantages provided by UHF‐MRI, certain drawbacks must also be taken into account. Commonly discussed disadvantages include some physiological considerations (more intensively pronounced unpleasant transient effects such as vertigo and nausea discomfort (Rauschenberg et al., 2014) but also some technical aspects. As field strength increases, field inhomogeneity—both in the local magnetic field (B0) due to increased magnetic susceptibility effects and in the radiofrequency transmit and receive fields (B1+ and B1−) due to dielectric effects—can cause image artifacts such as geometric distortion and image intensity biases over the brain (Polimeni, Renvall, Zaretskaya, & Fischl, 2018). This may particularly affect single‐shot echo‐planar imaging (EPI), which represents the mainly used application in fMRI imaging (Preibisch, Castrillón, Bührer, & Riedl, 2015). Besides geometrical distortions, the technical challenges further include position‐dependent flip angle, poor inversion, unexpected contrast, intravoxel dephasing as well as increased tissue‐specific absorption rates (SAR) and susceptibility‐induced magnetic field variations within a region of interest (ROI; Ladd et al., 2018). The search for adequate solutions to the issues mentioned above has progressed but is far from being complete (Düzel, Costagli, Donatelli, Speck, & Cosottini, 2021; Ladd et al., 2018).

One of the most important requirements for valid biomarkers is reproducibility (Strimbu & Tavel, 2010). In this context, numerous studies have investigated the reliability of RS measurements using MRI at 1.5 and 3.0 Tesla (Braun et al., 2012; Klomp et al., 2013; Manoach et al., 2001; Plichta et al., 2012; Q. Zou et al., 2015) and have demonstrated reproducible results. For example, Somandepalli and colleagues examined reliability within and across diagnostic groups of children with attention‐deficit/hyperactivity disorder and typically developing children (Somandepalli et al., 2015). They also examined voxel‐wise reliability between groups. Their results demonstrated moderate‐to‐high reliability across all children and within groups and additionally found that the higher‐order functional networks showed more than the lower one (Somandepalli et al., 2015). Z. Li, Kadivar, Pluta, Dunlop, and Wang (2012) examined the reproducibility of different fMRI matrices, such as seed region‐based FC, regional homogeneity (ReHo), and the amplitude of low‐frequency fluctuation (ALFF), in the RS brain and demonstrated the test–retest reproducibility for ReHo and ALFF in the whole gray matter.

Moreover, long‐term reproducibility studies have also shown good results. Song, Panych, and Chen (2016) demonstrated that substantial to moderate long‐term within‐subject reproducibility can be achieved in rs‐fMRI by applying data‐driven and predefined ROI‐based quantification of reproducibility. Chou, Panych, Dickey, Petrella, and Chen (2012) also examined the long‐term reproducibility of intrinsic connectivity networks and reported that RS intrinsic connectivity network parameters might be appropriate biomarkers for monitoring disease progression and treatments.

Although studies relating to the stability of FC measurements at standard field strengths are relatively abundant, far fewer have been conducted at UHF. Recently, Geissberger et al. (2020) investigated the reproducibility of amygdala activation in facial emotion processing at 7 Tesla and found fair to good intersession reliability and excellent reliability for averages over runs. In another study, Berboth, Windischberger, Kohn, and Morawetz (2021) investigated the voxel‐wise test–retest reliability of brain activity in response to an emotion regulation task for predefined ROIs implicated in four neural networks. Although test–retest reliability varied considerably across the emotion regulation networks and respective ROIs, high reliability was found in core emotion regulation regions, including the ventrolateral and dorsolateral prefrontal cortex (vlPFC and dlPFC) as well as the middle temporal gyrus (MTG).

Finally, Branco, Seixas, and Castro (2018) used a publicly released data set from the consortium for reliability and reproducibility (Zuo & Xing, 2014) to examine the temporal reliability of the sensorimotor and language networks. The authors reported good temporal reliability at short and medium time scales, as demonstrated by high values of overlap in the same session and 1 week after, for both networks. The results were also shown to be stable, irrespective of data quality metrics and physiological variables.

Given the paucity of research into the reliability of the properties of the core RS networks at UHF, this study aims to address this issue with a focus on three established RS networks—the default mode network (DMN), the central executive network (CEN), and the salience network (SN)—often subsumed as the triple‐network model (TNM). The networks of the TNM are considered to be the core of neurocognitive networks due to the involvement in a wide range of cognitive tasks (Menon & Uddin, 2010). Moreover, disruption in the synchronized activity of the triple networks has been implicated in various psychiatric diseases (Dong, Wang, Chang, Luo, & Yao, 2017; Jiang et al., 2017; C. Li et al., 2019; Menon, 2011) that often show overlapping dysfunctions particularly in those three networks. In the meantime, it is widely accepted that coordination of these networks plays a key regulatory role in organizing neural responses underlying fundamental brain functions (Nekovarova, Fajnerova, Horacek, & Spaniel, 2014) and it has been proposed that a deepening of the knowledge considering the TMN may be essential to understand pathophysiological dysfunction across several psychiatric disorders, as dysfunction in one network may affect the other two (Menon, 2011).

Thus, we chose to use the publicly available data set obtained from the Gorgolewski project (Gorgolewski et al., 2015) to investigate the stability of these three core RS networks in terms of the inter‐session stability of the fMRI parameters and the stability of the inter‐network correlations between the triple networks. The following four fMRI parameters were used to characterize the different properties of the brain networks: both ALFF (Yu‐Feng et al., 2007) and fractional ALFF (fALFF; Q.‐H. Zou et al., 2008) were used to evaluate the regional spontaneous activity. Specifically, ALFF indicates the strength of regional spontaneous brain activity, while fALFF represents the relative contribution of specific low‐frequency fluctuation to the whole frequency range. The ReHo (Zang, Jiang, Lu, He, & Tian, 2004) was used to investigate local FC; and the degree centrality (DC; Zuo et al., 2012), was used to investigate global FC. Thus, ReHo and DC are considered to be mutually complementary for detecting both local and remote brain activity synchronization (Cui et al., 2016). Together with the ALFF and fALFF parameters, these fMRI metrics enable comprehensive rsNW characterization, displaying a pattern of RS activity, regional temporal integration, and connectivity.

## 2 METHODS

### 2.1 Subjects

The data used in this study were taken from the open‐access data set (Gorgolewski et al., 2015). The original data set consisted of 22 healthy subjects (12 male and 10 female). We excluded six subjects due to head motion and technical issues. The exact reasons for the exclusion are given in Table S1. Therefore, our final data set originated from 16 subjects (9 male and 7 female) age range 22–29 years; mean 25.25 ± 2.01. According to the original publication, all subjects signed written informed consent. The study was performed in accordance with the declaration of Helsinki and was approved by the Ethical Committee of the Leipzig University.

### 2.2 Experimental procedure

The full experimental procedure is described in the original publication of Gorgolewski et al. (2015). All subjects were examined twice using a 7‐Tesla whole‐body MRI scanner (MAGNETOM 7 Tesla, Siemens Healthcare, Erlangen, Germany). The time between the two sessions was 1 week. Both examinations involved RS fMRI measurements. The focus of our investigation is on the results reported from the 15‐min RS sessions.

### 2.3 MR data acquisition

During the RS scanning sessions, the subjects were asked to remain awake, keep their eyes open, and focus on a cross. The subjects were also asked to abstain from drinking caffeinated products for at least 2 h before each scan.

All imaging protocols are presented in the original publication of Gorgolewski et al. (2015). MR data were acquired using a 7‐Tesla whole‐body scanner (MAGNETOM 7 T, Siemens Healthcare, Erlangen, Germany). A combined transmit receive head coil (consisting of a birdcage transmitter and 24 channels phased array receiver; NOVA Medical Inc, Wilmington, MA, USA) was used for imaging. The fMRI data were acquired using an EPI two‐dimensional sequence. Data were acquired in the axial orientation. Three hundred volumes were acquired in 15 min for each RS run with the following parameters: repetition time (TR) = 3,000 ms, echo time (TE) = 17 ms, partial Fourier 6/8, GRAPPA acceleration factor iPAT = 3, flip angle (FA) = 70°, field‐of‐view (FOV) = 192 × 192 mm, imaging matrix 128 × 128 × 70 slices, slice thickness = 1.5 mm, and voxel size 1.5 mm^3^.

High‐resolution T1‐weighted images were acquired using a three‐dimensional magnetization prepared rapid gradient echo (3D MP‐2RAGE) sequence (TR = 5 s, TE = 2.45 ms, TI_1/2_ = 0.9/2.75 s, partial Fourier 6/8, GRAPPA acceleration factor iPAT = 2, FA_1/2_ = 5°/3°, FOV = 224 × 224 × 168 mm^3^, imaging matrix 320 × 320 × 240, and voxel size 0.7 mm^3^).

### 2.4 fMRI data preprocessing and analysis

The fMRI images were preprocessed using data processing and analysis for brain imaging (Yan, Wang, Zuo, & Zang, 2016) and SPM12 (http://www.fil.ion.ucl.ac.uk/spm/) toolboxes built on MATLAB software package version 2017b (The Math Works, Inc., Natick, MA, USA). The preprocessing was performed as follows: the first 10 volumes were removed, followed by slice timing correction, realignment of images and field map correction, individual T1 images were co‐registered to the functional images. The transformed T1 images were segmented to grey matter, white matter (WM), and cerebrospinal fluid (CSF). Then the Friston 24‐parameter model was used to remove the nuisance signals by regressing out the head motion effects from the realigned data. Also, the signals from WM and CSF were regressed out to reduce the impact of physiological noise. As motion could influence the FC results, the Friston 24‐parameter model and framewise displacement were used to estimate any head motion at the subject level. Any subjects who had head motion exceeding 1.5 mm in translation or 1.5° in rotation were excluded (Table S1). Afterward, to keep only the high‐quality data, motion scrubbing was applied to remove minimal motion frames (volumes exhibiting framewise displacement >0.2 mm were excluded). After scrubbing, the percentage of volumes left for each subject and session is reported in Table S2. Then the fMRI parameters were computed in the native space. Default masks (whole brain, white matter, gray matter, and CSF) were generated based on the segmented T1 image and then were applied before computing the fMRI parameters. ALFF value was calculated by transforming the BOLD signal time series to the frequency domain using the fast Fourier transformation, then the power spectrum was obtained. Later ALFF is calculated as the sum of amplitudes within a low‐frequency band of 0.01–0.1 Hz for each voxel (Yu‐Feng et al., 2007). The fALFF value was calculated dividing the power within the low‐frequency range (ALFF) by the total power in the entire measurable frequency range (Zuo et al., 2010). Later temporal filtering between 0.01 and 0.1 Hz was applied to all voxels time series on the preprocessed fMRI data to calculate DC and ReHo. The DC was computed by calculating Pearson's correlation coefficient between the time series of a given voxel and all other gray matter voxels in the brain. The correlation vector was binarized by applying a threshold (*r* > .25, *p* ≤ .001) and added (Takeuchi et al., 2015). The ReHo was computed by averaging the synchronization or similarity between the time series of a given voxel and its 26 neighboring voxels using Kendall's coefficient of concordance (Zang et al., 2004). The fMRI parameters were normalized using a *Z*‐score standardization procedure (subtracting the mean from each voxel and then dividing the value by the *SD* of the whole brain). Finally, spatial smoothing with full width at half maximum (FWHM) at 3 mm^3^ was applied.

### 2.5 Triple‐network ROIs

**FIGURE 1:**
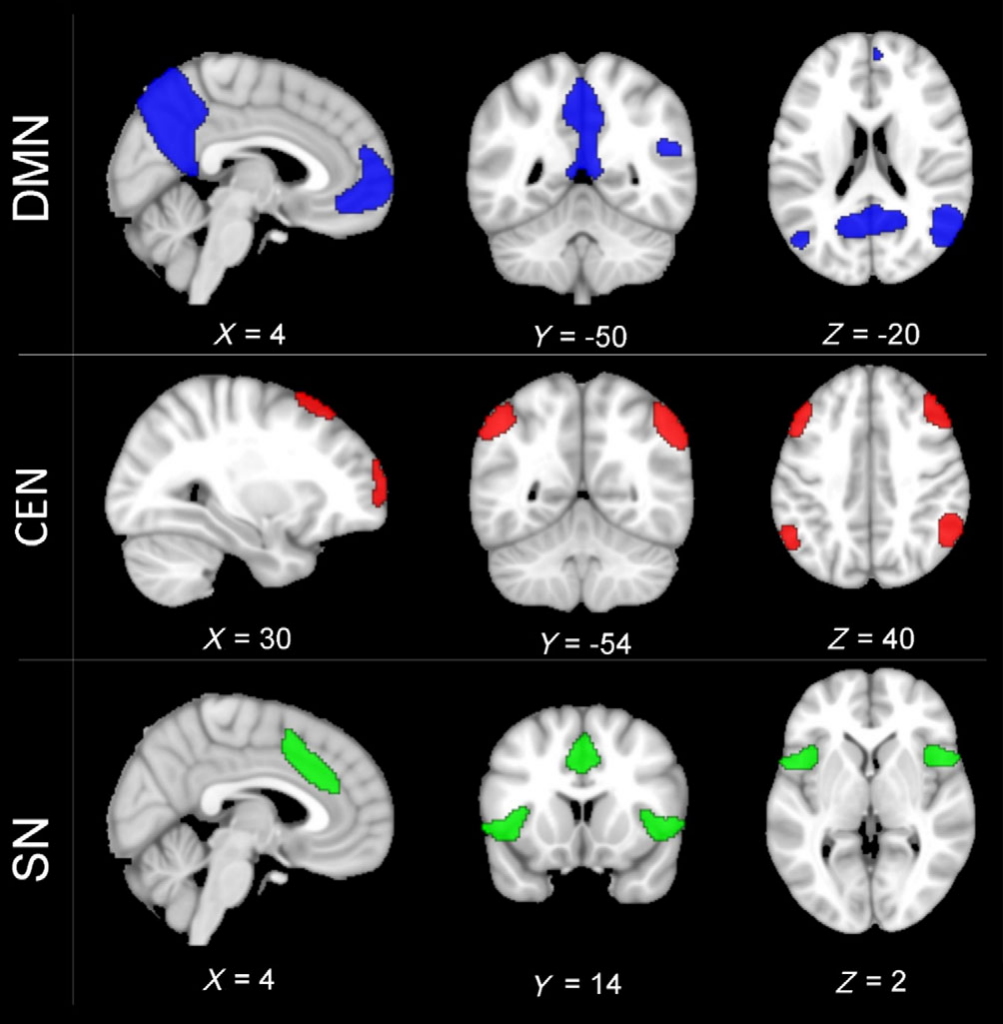
Depiction of the masks for the three core resting state networks: the default mode network (DMN, blue color), the central executive network (CEN, red color), and the salience network (SN, green color) overlaid on MNI152 template. CEN, central executive network; DMN, default mode network; SN, salience network

The publicly available data set was used to specifically extract and analyze the triple RS networks. The TNM included 15 ROIs, which were selected following the specifications from networks atlas provided by the Conn toolbox (Whitfield‐Gabrieli & Nieto‐Castanon, 2012). The masks for the DMN and CEN consisted of four ROIs each, and the SN mask included seven ROIs. Concretely, the DMN included the medial prefrontal cortex (MPFC), the left and right lateral parietal cortex and the posterior cingulate cortex (PCC). The CEN included the right and left lateral prefrontal cortex (rPFC, lPFC) and the right and left posterior parietal cortex (PPC). The SN included the anterior cingulate cortex (ACC), the left and right anterior insula, the left and right rostral prefrontal cortex, and the left and right supramarginal gyrus. The masks for the three networks are shown in Figure 1.

### 2.6 ROI‐based FC analysis

To keep the original spatial resolution of the functional images, the defined masks were warped into the individual space, and then the connectivity between the ROIs within the triple networks was computed by extracting the mean BOLD signal time series from each of three networks' ROI. Pearson's correlation coefficient was computed between each pair of the ROI's mean BOLD signal time series, resulting in two 15 × 15 connectivity matrices for each subject. Fisher's *r* to *z* transformation was applied for all connectivity matrices to improve the normality.

### 2.7 Stability analysis

#### 2.7.1 Intersession stability of the fMRI parameters in the triple networks

We used Lin's concordance correlation coefficient (CCC) instead of Pearson's correlation because it estimates the variation from the 45° line (L. I. Lin, 1989; McBride, Lin, Bland, & Altman, 2005), thus, the CCC gives more accurate reliability results. Lin's CCC ($\rho_{c})$ was calculated using the following formulas (1–8) (L. I. Lin, 1989; L. Lin, Hedayat, Sinha, & Yang, 2002; McBride et al., 2005):

(1) $$\rho_{c}=\frac{2\sigma_{\mathrm{yx}}}{\begin{array}{c} \;\\ \;{\sigma_{y}}^{2}+{\sigma_{x}}^{2}+{\left(\mu_{y}-\mu_{x}\right)}^{2} \end{array}},$$

where the mean ($\mu_{x}$) of each fMRI parameter in each session was computed as follows:

(2) $$\mu_{x}=\frac{1}{n}\sum_{n=1}^{n}\;x_{n}.$$

The variance ($\sigma_{x}^{2}$) within each session for each fMRI parameter was computed as follows:

(3) $$\sigma_{x}^{2}=\frac{1}{n}\sum_{n=1}^{n}{\left(x_{n-}\mu_{x}\right)}^{2}.$$

The covariance ($\sigma_{\mathrm{yx}}$) between two sessions for each fMRI parameter was computed as follows:

(4) $$\sigma_{\mathrm{yx}}=\frac{1}{n}\sum_{n=1}^{n}\left(y_{n}-\mu_{y}\right)\left(x_{n}-\mu_{x}\right).$$

The CCC can also be written as a product of accuracy and precision $\rho_{c}=\chi_{a}\rho$, where precision ρ is Pearson's correlation coefficient and accuracy is the term $\chi_{a}$ given by the equation:

(5) $$\chi_{a}=\frac{2}{\varpi +\frac{1}{\varpi }+v^{2}},$$

where

(6) $$v^{2}=\frac{{\left(\mu_{y}-\mu_{x}\right)}^{2}}{\sigma_{y}\sigma_{x}},$$

and

(7) $$\varpi =\frac{\sigma_{y}}{\sigma_{x}}.$$

Also, the sample counterpart of CCC is given by

(8) $$r_{c}=\frac{2rS_{y}S_{x}}{\begin{array}{c} \;\\ \;{S_{y}}^{2}+{S_{x}}^{2}+{\left(\bar{y}-\bar{x}\right)}^{2} \end{array}},$$

where the r is sample Pearson's correlation coefficient, $\bar{y}\;\text{and}\;\bar{x}$ are the sample means, and the $\begin{array}{c} \;\\ \;{S_{y}}^{2}\;\text{and}\;{S_{x}}^{2} \end{array}$ are the sample variances.

The four fMRI parameters (ALFF, fALFF, ReHo, and DC) were extracted from the triple‐network voxels for all subjects in both sessions. The extracted values were used to calculate the voxel‐based CCC at the subject level. CCC was calculated using MATLAB‐based function f_CCC available at https://github.com/robertpetermatthew/f_CCC/blob/master/f_CCC.m (Robert Matthew 2020). CCC including the confidence interval for the same was calculated with an adjusted significance threshold (*α*) of .00026. The significance threshold was precomputed accounting for multiple comparison correction via Bonferroni method (Chen, Feng, & Yi, 2017). Since 16 subjects were tested for four fMRI parameters in three RS networks, a total of 192 tests were made. This resulted in adjusted significance threshold (*α*) of .05/192 = .00026. The computed CCC value and the confidence intervals with adjusted alpha are reported in Table S3.

The mean of the intermeasurement stability for the fMRI parameters in each network was calculated. Thereby, the Dancey and Reidy scale was applied to interpret Pearson's and Spearman's correlation coefficients (Dancey & Reidy, 2004). Thus, correlation coefficients <.40 were considered to be weak, values between .40 and .69 were considered moderate, values between .70 and .99 were considered strong, and correlation coefficients of 1.00 were considered perfect. As the CCC should be interpreted close to other correlation coefficients (e.g., Pearson's; Altman & Altman, 1999; Akoglu, 2018), we used the same scale for assessing both the inter‐session stability of the fMRI parameters and the stability of the internetwork correlations.

#### 2.7.2 Stability of the internetwork correlations between the three networks

**FIGURE 2:**
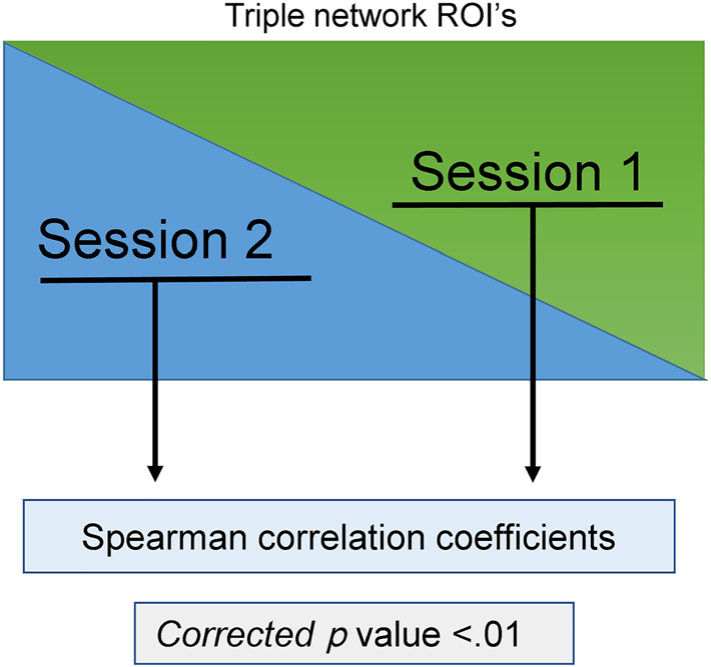
Schematic representation of the calculation procedure for assessing internetwork stability. We first calculated the connectivity between each network pair in each session, and Spearman's correlation coefficients were then calculated from those values to determine the inter‐session stability of the internetwork connectivity

To calculate the stability of the FC between the three networks, the internetwork connectivity matrices of the triple networks were computed for each session. Subsequently, Spearman's correlation coefficients between the connectivity values in each pair of networks, in each session, at a significance level of *p* < .01 were calculated as shown in Figure 2. False discovery rate (FDR) was used to correct for multiple comparison. The mean value of Spearman's correlation coefficients for each pair of networks was then calculated. Spearman's correlation was used instead of Pearson's correlation owing to the small sample size (4, 4, and 7 ROIs in the DMN, CEN, and SN, respectively).

## 3 RESULTS

### 3.1 Intersession stability of the fMRI parameters in the triple networks

**FIGURE 3:**
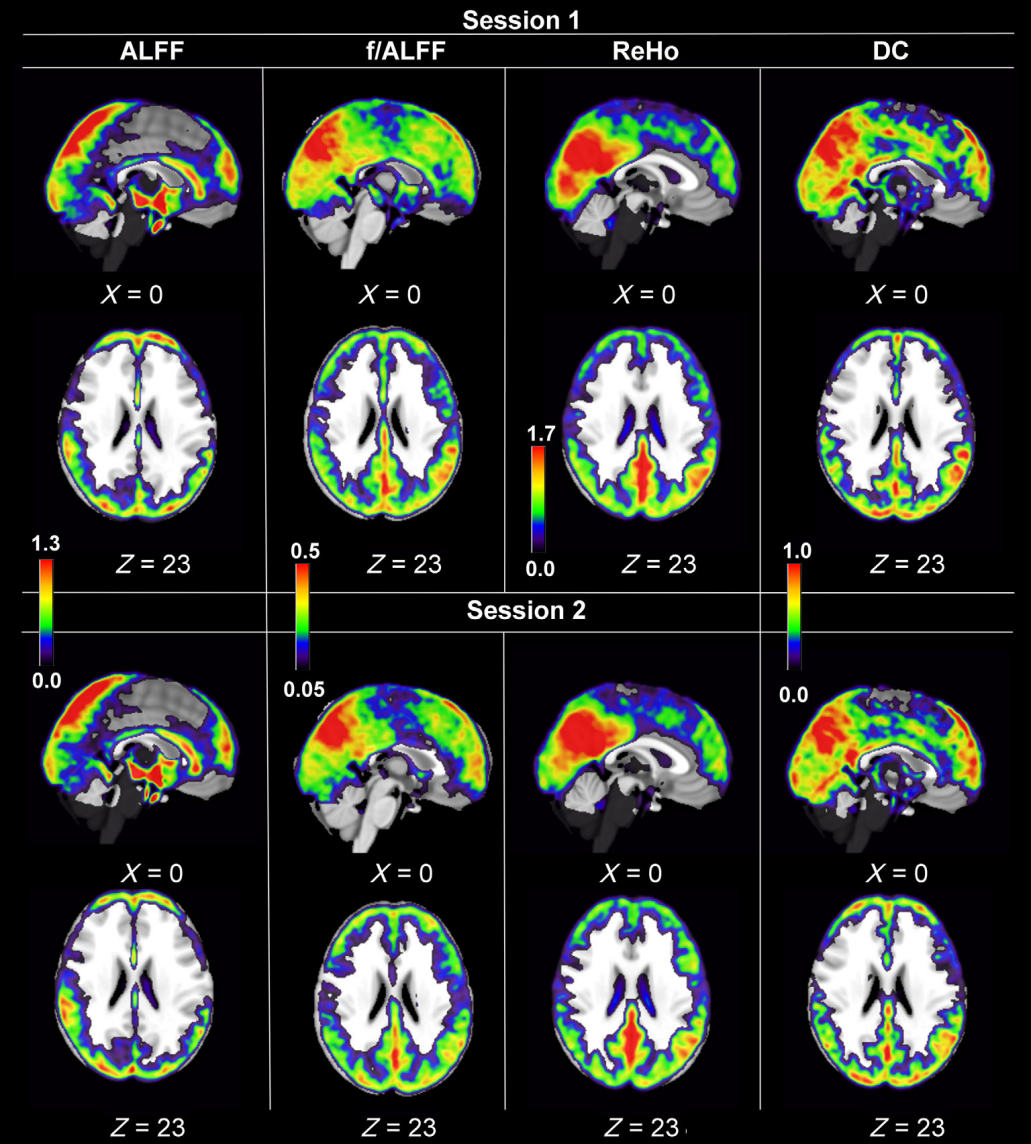
Depiction of the average of each functional magnetic resonance imaging (fMRI) parameters (amplitude of low‐frequency fluctuations [ALFF], fractional ALFF [fALFF], regional homogeneity [ReHo], and degree centrality [DC]) as computed from 16 subjects in both sessions. All analysis took place in the native space. Only for presentation purposes, the values were transferred to the standard space. The color bar is common for both sessions in ReHo parameter

The mean values of the fMRI parameters (ALFF, f/ALFF, ReHo, and DC) across 16 subjects obtained in each session are visualized in Figure 3. The visual inspection reveals stable levels of ALFF and fALFF) as well as DC and ReHo.

The values of the CCCs, representing the intraindividual, intermeasure stability of the fMRI parameters for each participant in each network, are shown in Table 1.

**TABLE 1:** The intraindividual intermeasure stability of the spontaneous brain activity and connectivity (short and long) within the triple networks represented by concordance correlation coefficients. The gray highlight indicates the computed CCC value and the confidence intervals with adjusted alpha (0.00026) are reported in Supplementary Material ‐ Table 3 (S‐Tab. 3).

Subjects		DMN				CEN				SN		
	ALFF	fALFF	ReHo	DC	ALFF	fALFF	ReHo	DC	ALFF	fALFF	ReHo	DC
Sub01	0.72	0.19	0.77	0.40	0.75	0.02	0.75	0.25	0.74	−0.20^a^	0.70	0.24
Sub02	0.93	0.76	0.87	0.75	0.88	0.69	0.88	0.73	0.92	0.67	0.89	0.81
Sub03	0.84	0.44	0.64	0.47	0.69	0.44	0.56	0.62	0.87	0.35	0.76	0.78
Sub04	0.93	0.80	0.92	0.74	0.85	0.62	0.89	0.80	0.91	0.66	0.88	0.84
Sub05	0.76	0.74	0.93	0.87	0.83	0.52	0.84	0.80	0.76	0.41	0.84	0.81
Sub06	0.96	0.70	0.90	0.61	0.90	0.51	0.84	0.65	0.93	0.37	0.85	0.67
Sub07	0.90	0.61	0.84	0.76	0.85	0.56	0.82	0.77	0.90	0.41	0.79	0.64
Sub08	0.93	0.62	0.81	0.71	0.90	0.50	0.73	0.70	0.95	0.46	0.79	0.64
Sub09	0.96	0.85	0.91	0.89	0.94	0.90	0.94	0.90	0.92	0.75	0.84	0.90
Sub10	0.93	0.75	0.92	0.83	0.92	0.63	0.90	0.71	0.95	0.58	0.81	0.67
Sub11	0.95	0.75	0.91	0.76	0.95	0.76	0.93	0.77	0.92	0.69	0.86	0.78
Sub12	0.89	0.59	0.89	0.75	0.87	0.61	0.91	0.67	0.90	0.59	0.87	0.78
Sub13	0.93	0.73	0.92	0.71	0.93	0.69	0.87	0.71	0.89	0.73	0.83	0.66
Sub14	0.91	0.72	0.91	0.72	0.81	0.72	0.80	0.73	0.90	0.75	0.69	0.87
Sub15	0.91	0.60	0.74	0.32	0.92	0.73	0.83	0.63	0.95	0.83	0.91	0.72
Sub16	0.95	0.68	0.93	0.88	0.91	0.64	0.89	0.88	0.95	0.61	0.91	0.92

*Note*: All correlation coefficients were significant except the fALFF in CEN for the first subject (potential outlier subject).

Abbreviations: ALFF, amplitude of low‐frequency fluctuation; CEN, central executive network; DC, degree centrality; DMN, default mode network; fALFF, fractional amplitude of low‐frequency fluctuation; ReHo, regional homogeneity; SN, salience network.

^a^ The marked subject is considered as a potential outlier; thus, a cross‐check of all the raw data and all technical issues mentioned in the original publication was performed. The cross‐check did not reveal any particularities for this subject (partial signal drop out or movement). However, when an additional analysis without this subject was performed, the results from this sample size (15 participants) did not differ noticeably from the findings from the whole sample (16 participants), as shown in Figures S1 and S2.

**FIGURE 4:**
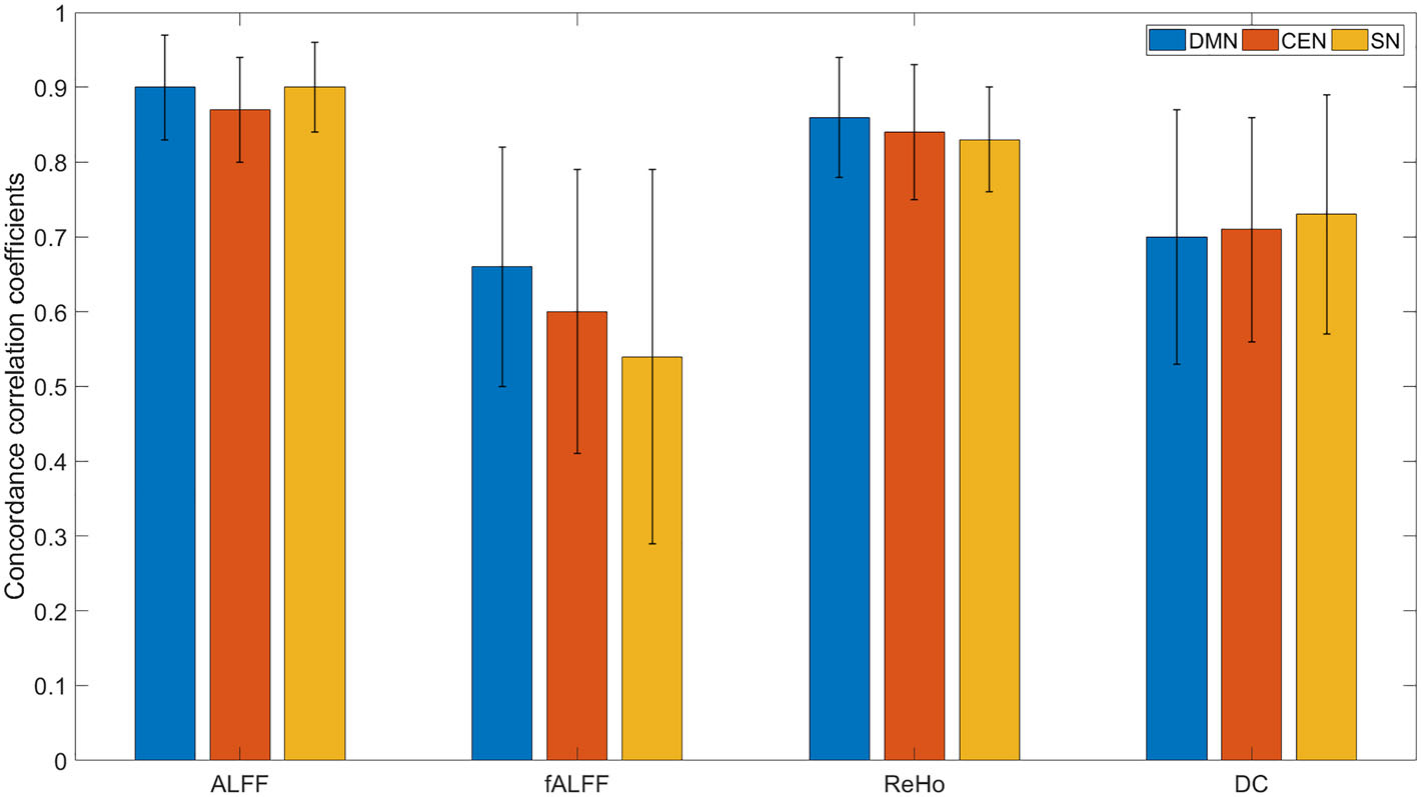
Mean of the inter‐measurement stability across 16 subjects for the fMRI parameters (amplitude of low‐frequency fluctuations (ALFF), fractional ALFF (fALFF), regional homogeneity (ReHo), and degree centrality (DC)) in each core network ‐ the default mode network (DMN), the central executive network (CEN), and the salience network (SN). The error bars represent standard deviation

Figure 4 illustrates the mean of the intermeasurement stability for the fMRI parameters in each network

We observed the highest mean stability of ALFF parameter to be in both the DMN (0.90 ± 0.07; range from 0.72 to 0.96; strong stability) and the SN (0.90 ± 0.06; range from 0.74 to 0.95; strong stability) closely followed by the CEN (0.87 ± 0.07; range from 0.69 to 0.95; strong stability).

The mean stability of fALFF parameter was moderate in all three networks: DMN: 0.66 ± 0.16 (range from 0.19 to 0.85); CEN: 0.60 ± 0.19 (range from 0.02 to 0.90); SN: 0.54 ± 0.25 (range from −0.20 to 0.83).

The mean stability of ReHo parameter was found to be strong in all three networks: DMN: 0.86 ± 0.08 (range from 0.64 to 0.93); CEN: 0.84 ± 0.09 (range from 0.56 to 0.94); SN: 0.83 ± 0.07 (range from 0.69 to 0.91).

The mean stability of DC parameter was found to be strong in all three networks: SN: 0.73 ± 0.16 (range from 0.24 to 0.92); CEN: 0.71 ± 0.15 (range from 0.25 to 0.90); DMN: 0.70 ± 0.17 (range from 0.32 to 0.89).

### 3.2 Stability of the internetwork correlations between the three networks

**FIGURE 5:**
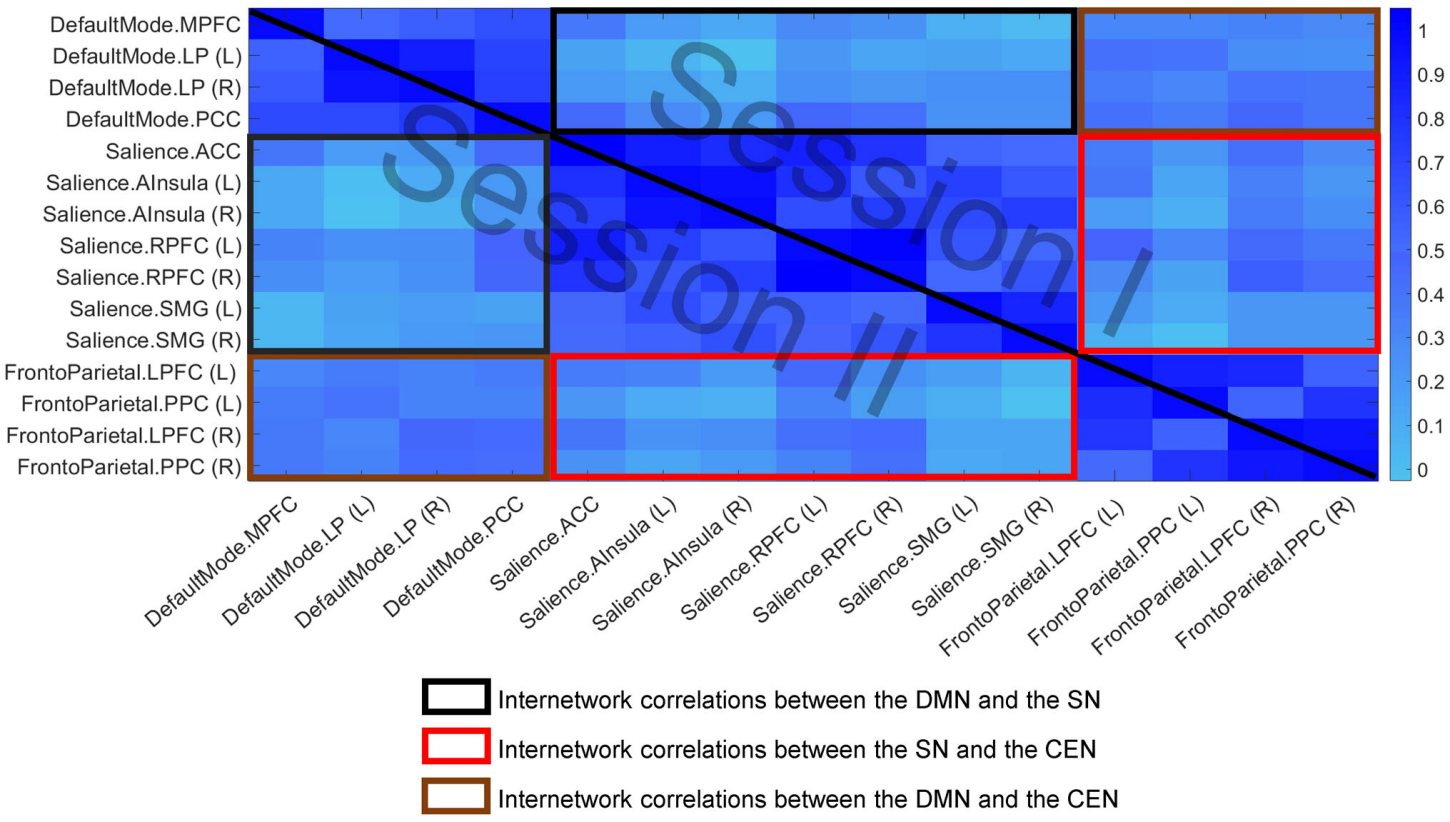
The functional connectivity average matrix for 16 subjects between the regions of the three core resting state networks. The default mode network (DMN) includes the following regions: the medial prefrontal cortex (MPFC), the left and right lateral parietal cortex (LP(L), LP(R), and the posterior cingulate cortex (PCC). The central executive network (CEN) includes the right and left lateral prefrontal cortex (rPFC, lPFC) and the right and left posterior parietal cortex (PPC). The salience network (SN) includes the anterior cingulate cortex (ACC), the left and right anterior insula (Insula), the left and right rostral prefrontal cortex (RPFC), and the left and right supramarginal gyrus (SMG). The upper triangular matrix represents the first session, and the lower triangular matrix represents the second session

**FIGURE 6:**
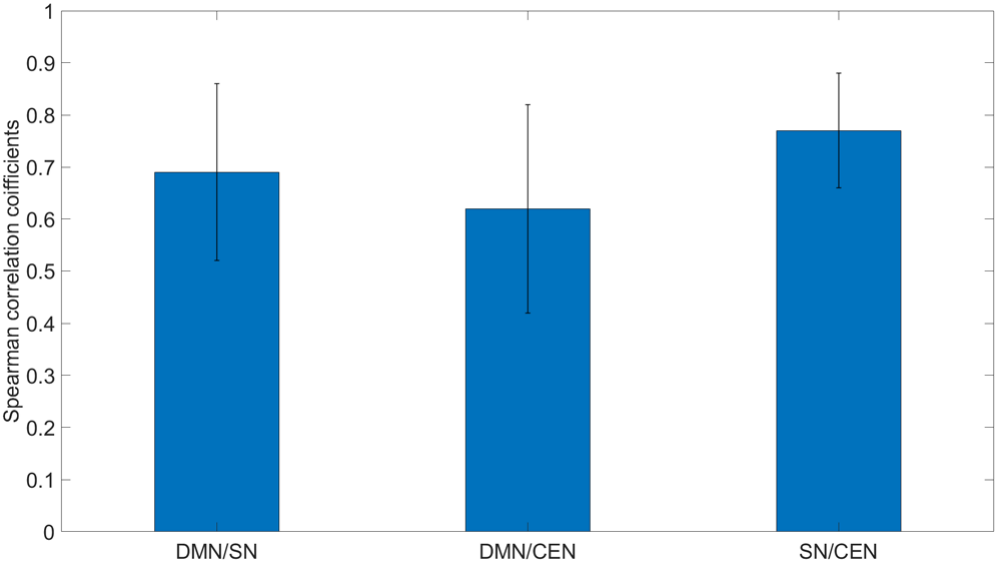
Mean of the internetwork correlations stability between the three core resting state networks depicted as the mean correlations between DMN/SN, DMN/CEN, and SN/CEN. CEN, central executive network; DMN, default mode network; SN, salience network

The ROI‐based FC correlation matrix obtained during each of the two measurements is shown in Figure 5, and the concrete values are given in the Tables S4 and S5. Spearman's correlation coefficients representing the stability of the internetwork FC between the triple networks at the individual level are shown in Table 2. Also, the mean values of Spearman's correlation coefficients for each pair of networks are shown in Figure 6.

**TABLE 2:** Spearman's correlation coefficients and the corresponding *p* values, which represent the stability of the functional connectivity between each pair of networks (DMN/SN, DMN/CEN, and SN/CEN) when compared between the two sessions for 16 subjects

Subjects	DMN/SN		DMN/CEN		SN/CEN	
	Spearman's correlation coefficients	Corrected *p* value	Spearman's correlation coefficients	Corrected *p* value	Spearman's correlation coefficients	Corrected *p* value
Sub01	.58	<.01	.71	<.01	.69	<.01
Sub02	.47	<.01	.61	<.01	.61	<.01
Sub03	.59	<.01	.53	<.01	.60	<.01
Sub04	.63	<.01	.76	<.01	.80	<.01
Sub05	.81	<.01	.79	<.01	.74	<.01
Sub06	.56	<.01	.12	<.01	.80	<.01
Sub07	.71	<.01	.69	<.01	.72	<.01
Sub08	.61	<.01	.82	<.01	.91	<.01
Sub09	.80	<.01	.60	<.01	.68	<.01
Sub10	.85	<.01	.69	<.01	.89	<.01
Sub11	.94	<.01	.94	<.01	.91	<.01
Sub12	.61	<.01	.59	<.01	.90	<.01
Sub13	.87	<.01	.41	<.01	.67	<.01
Sub14	.84	<.01	.78	<.01	.90	<.01
Sub15	.36	<.01	.34	<.01	.71	<.01
Sub16	.85	<.01	.60	<.01	.85	<.01

Abbreviations: CEN, central executive network; DMN, default mode network; SN, salience network.

The internetwork connectivity showed a strong to moderate stability between the investigated network pairs: between DMN and SN: 0.69 ± 0.17 (range from 0.36 to 0.94); between DMN and CEN: 0.62 ± 0.20 (range from 0.12 to 0.94); between CEN and SN: 0.77 ± 0.11 (range from 0.60 to 0.91). Bars represent standard deviation.

## 4 DISCUSSION

The aim of this study was to investigate the stability of the properties of three core RS networks (DMN, CEN, and SN) and the reliability of the connectivity between these networks based on the Conn Atlas (Whitfield‐Gabrieli & Nieto‐Castanon, 2012). The analysis was performed using a publicly available open‐access data set (Gorgolewski et al., 2015) obtained at a 7‐Tesla UHF‐MRI scanner. The study was motivated by the question of whether RS data from UHF examinations could be used for extraction of biomarkers to potentially support diagnosis as well as therapy planning and monitoring) in psychiatric disorders.

In terms of intersession stability, our investigation revealed CCCs of a moderate to strong level. Specifically, the strongest stability was observed for the ALFF parameter that showed a strong stability in all three networks. This observation confirms previous reports about the high test–retest reproducibility of different parameters characterizing the spontaneous brain activity obtained at standard field strengths (Jia et al., 2020; Z. Li et al., 2012; Somandepalli et al., 2015; Zuo, Biswal, & Poldrack, 2019; Zuo & Xing, 2014). Generally, the ALFF reflects spontaneous neural activity of the brain (Zuo et al., 2010). In recent years, ALFF has been increasingly applied to characterize neuropsychiatric disorders, such as attention deficit and hyperactivity disorder (Zang et al. 2007), Alzheimer's disease (He et al., 2007), mild cognitive impairment (Han et al., 2012), bipolar disorder (J. Liu et al., 2012), schizophrenia (Hoptman et al., 2010; Turner, 2013), Tourette's syndrome (Cui et al., 2014) and obsessive–compulsive disorder (Bu et al., 2019).

In parallel to ALFF, we also analyzed the stability of fALFF, which is considered as an improved approach to ALFF detection (Q.‐H. Zou et al., 2008), and in particular measures the relative contribution of low‐frequency fluctuations within a specific frequency band with respect to the whole detectable frequency range (Zuo et al., 2010). Thus, it enables the amplitude of regional neuronal activity to be studied, potentially identifying brain areas with abnormal local functioning (Chen et al., 2015). In our study, the stability of the fALFF values was moderate in all three networks. This somewhat lower stability of fALFF compared with ALFF is in concordance with come previous reports (Küblböck et al., 2014; Yan, Craddock, Zuo, Zang, & Milham, 2013; Zuo et al., 2010). While ALFF is more receptive for the potential artifactual in the neighborhood to blood vessels and the cerebral ventricles, fALFF is a proportional parameter composed of ALFF in the numerator and the sum of the amplitudes in the whole frequency spectrum in the denominator (Küblböck et al., 2014) Thus, a decrease in reliability of their ratio is to be expected (Arndt, Cohen, Alliger, Swayze II, & Andreasen, 1991).

The SN showed somewhat lower (still moderate) level of fALFF stability compared to the DMN and the CEN. The SN is involved in detecting, filtering, and integrating relevant internal (e.g., autonomic input) and external (e.g., emotional information) salient stimuli to guide behavior (Bressler & Menon, 2010; Chand & Dhamala, 2016). Furthermore, it has a crucial role in the functional and dynamic switching between the DMN and CEN (i.e., between task‐based and task‐free states; Y. Liu et al., 2017; Zheng et al., 2015). Thereby, the SN responds to the subjective degree of salience (Goulden et al., 2014). It is thought that different regions of the SN could form a sort of information processing loop for representing and responding to homeostatically relevant external and internal stimuli (Seeley, 2019). Indeed, their involvement in emotional functions (Heimer & Van Hoesen, 2006), autonomic functions, and self‐awareness (Craig, 2002), as well as in the process of internal (self‐)reference that predominate in states of rest and disengagement (Critchley, 2005), is well documented. Since all these internal stimuli can hardly be kept constant at different measurement times (despite the uniform absence of special tasks), it is not surprising that the stability of regional spontaneous activity is somewhat lower in the SN than in the other two networks.

We observed further for all three networks a strong stability for the parameter ReHo. Often designated as a local FC, ReHo defined by the temporal coherence or synchronization of the BOLD time series within a set of a given voxel's nearest neighbors (Jiang & Zuo, 2016) and is becoming increasingly recognized as being a highly sensitive and reliable neuroimaging marker to characterize the human brain (Jiang & Zuo, 2016). The high test–retest reliability of ReHo has been already confirmed in various studies, including a systematic analysis based on previously published papers (Zuo & Xing, 2014). However, the basis for this investigation was data acquired using standard field strength MRI. It has been shown that altered ReHo values may relate to disequilibrium in spontaneous neural activity within and between corresponding brain regions (Chen et al., 2015). Indeed, aberrant ReHo values, indicative of disrupted local functionality, have been linked to several neurological and psychiatric disorders, such as Alzheimer's disease (He et al., 2007), chronic pain attention‐deficit hyperactivity disorder (de Celis Alonso et al., 2014), autism spectrum disorders (Paakki et al., 2010), depression (Guo et al., 2011), bipolar disorder (Shan et al., 2020) and schizophrenia (Ma et al., 2019; Mwansisya et al., 2017); as well as in first‐degree relatives of patients with schizophrenia (Liao et al., 2012). In addition, a recent study demonstrated a significant association between ReHo in the DMN and resilience, as well as with the personality traits extroversion (in the CEN and SN) and conscientiousness (in the SN) (Altinok et al., 2021). Therefore, ReHo appears to be a valuable and powerful tool for detecting aberrant RS brain activity, which can be associated with a wide range of psychopathological abnormalities as well as with different personality traits and varying levels of resilience in healthy individuals.

The long‐connectivity parameter (DC) also showed a strong level of stability in all three networks, although the values of intermeasurement stability were somewhat below the values for ALFF and ReHo in all three networks. Divergent from our findings, previous studies reported considerably lower test–retest reliability of the long‐range connectivity compared to the short‐range connectivity parameters (Braun et al., 2012; Holiga et al., 2018; Telesford et al., 2010). In a comprehensive meta‐summary from previously published papers on the test–retest reliability of voxel‐wise metrics from non‐UHF studies, Zuo and Xing (2014) investigated the long‐term (∼6 months) test–retest reliabilities measured as intraclass correlation (ICC). In this study, the ICC values for DC ranged from approximately 0.25 in the limbic network (that includes parts of the SN) to 0.5 in the dorsal attentional network (that includes parts of the CEN). In the DMN and the control network, the ICC values reached a value of 0.4. Similarly, in a more recent study performed at a 3‐Tesla MRI scanner; the highest part of the observed voxel had a mean ICC value of about 0.35. In opposite to these reports, we obtained reliability for DC which has exceeded the value of 0.7 in all three networks examined. Our results indicate that, especially when investigating long‐distance connectivity, the use of 7‐Tesla MRI scanners could provide results that are more reliable and thus more reproducible than results from standard field strength MRI. Similar to our findings, one other recent work reports a significant increase of the test–retest reliability at the intranetwork when comparing seed‐based connectivity between 7 and 3 Tesla scans (Nemani & Lowe, 2021).

In our final analysis, we investigated the stability of the internetwork connectivity between each pair of the three core RS networks. Earlier investigations indicate that a stabile synchronization of these three networks plays a crucial role in higher cognitive functions. Thereby, the functional connectivity between the DMN and the SN appears to be important for cognitive control (Bonnelle et al., 2012; Menon & Uddin, 2010), and the SN has a central role in switching between the DMN and the CEN (Bonnelle et al., 2012; Liang, Zou, He, & Yang, 2015; Menon & Uddin, 2010; Seeley, 2019). Accordingly, aberrations in these intrinsically well‐organized interactions have been linked to pathological states with impaired cognition (Wang et al., 2015), and the observation of altered internetwork interactions generally may be a valuable indicator of psychiatric symptoms.

In our study, the subregions of the DMN and the SN showed predominantly weak positive correlations. Moderate correlations included in both sessions the PCC, showing correlations in the range of .42–.48 with three subregions of the SN (the ACC and the left and right RPFC). Regarding the synchronization between the DMN and the CEN, the correlations between the subregions were also mainly weak. We observed the strongest (moderate) correlations for PCC (with left and right LPFC in Session 1 and with the left LPFC and right PPC in Session 2), for left LP (with left LPFC in Session 1 and with left PPC in Session 2) as well as for the right LP (with right LPFC in Session 1 and with right LPFC and PPC in Session 2).

In terms of the association between the SN and the CEN, the SN subregions with the strongest positive associations with the CEN were the RPFC, with the moderate correlations observed between the RPFC(R) and the LPFC(R) in both sessions as well as moderate correlations between the left RPFC and left and right LPFC.

The relative strength of the correlations between the specific subregions remained widely constant when comparing the first and the second sessions.

At the whole network level, the internetwork connectivity showed a strong to moderate stability between the investigated network pairs. This high reliability of the between‐network connectivity is consistent with the latest report by Nemani and Lowe (2021), who, however, also showed that the high between‐network reliability at 7 Tesla was not significantly improved compared to the 3 Tesla results.

## 5 CONCLUSION

More than 20 years since the first UHF‐MRI scanners were approved for use in human (Robitaille et al., 1999; Yacoub et al., 2001), results from various areas of medicine demonstrate numerous unambiguous advantages of using this technology (Düzel et al., 2021; Platt et al., 2021; Vachha & Huang, 2021). Nevertheless, numerous technical challenges still need to be solved so that the applicability remains limited, especially in the field of clinical research (Düzel et al., 2021; Ladd et al., 2018). In this context, especially considering the growing need for the identification of reliable biomarkers for mental and neurological diseases, the issue of reproducibility of results remains crucial (Griffanti et al., 2016). In a recent work, a significantly increased reliability of RS connectivity at UHF strengths over conventional field strengths has been demonstrated (Nemani & Lowe, 2021). We complement this report with our confirmation of a strong stability of the three RS fMRI metrics (ALFF, ReHo and DC), representing the spontaneous brain activity, local‐ and long‐range connectivity, respectively, in three major RS networks. In opposite to previous investigations at standard field strengths, our results have also revealed a strong reliability for DC in all three networks examined. Thereby, DC could be considered a measure of the long‐distance connectivity and even more, it appears to be a very suitable measure to explore RS whole‐brain neural network connectivity, due to the reduction of the possible bias caused by selecting brain regions according to the priori assumption (Guo et al., 2020; Buckner et al., 2009; Zuo et al., 2012). Our observation suggests that for rsFMRI measurements at 7 Tesla, long‐range connectivity can also be considered a reliable parameter. Taking into account all other advantages of UHF imaging, this technology appears to be well suited for a versatile, reliable, and reproducible characterization of RS networks and their interactions, as shown here with the example of the three RS networks when investigating the regional brain activity, short and long connectivities.

## CONFLICT OF INTEREST

The authors declare no potential conflict of interest.

## AUTHOR CONTRIBUTIONS

**Hasan Sbaihat:** Data analysis, writing—original draft, methodology; **Ravichandran Rajkumar:** Methodology, software programming, draft review, and editing; **Shukti Ramkiran:** Methodology, draft review, and editing; **Abed Al‐Nasser Assi:** Supervision, draft—review and editing; **Jörg Felder:** discussion methodology, review, and editing; **N. Jon Shah:** Supervision, discussion and revision preprocessing, draft review, and editing. **Tanja Veselinović:** Methodology, supervision, draft review and editing; **Irene Neuner:** Conceptualization, supervision, funding, draft review, and editing.

## Supporting information


**FIGURE S1** Mean of the intermeasurement stability across 15 subjects (calculated from the sample excluding one potential outlier subject) for the fMRI parameters (amplitude of low‐frequency fluctuations [ALFF], fractional ALFF [fALFF], regional homogeneity [ReHo], and degree centrality [DC]) in each core network, that is, the default mode network (DMN), the central executive network (CEN), and the salience network (SN). The error bars represent *SD*.

**FIGURE S2** Mean of the internetwork correlations stability between the three core resting‐state networks depicted as the mean correlations between the DMN/SN, the DMN/CEN, and the SN/CEN. The error bars represent *SD*. This calculation was performed on the sample of 15 participants, that is, without the one participant who was considered as a potential outlier.

**TABLE S1** Showing the included and excluded subjects in our study. Some subjects were excluded due to motion, missing of the data, or low‐resolution fMRI images.

**TABLE S2** Showing the percentage of volumes left for each subject and session after scrubbing.

**TABLE S3** The concordance correlation coefficients and their 95 confidence interval level of significance for the fMRI parameters between the two sessions in the default mode network (DMN), the central executive network (CEN), and the salience network (SN).

**TABLE S4** The average values of the inter‐network interaction of the default mode network (DMN), the central executive network (CEN), and the salience network (SN) in Session 1.

**TABLE S5** The average values of the internetwork interaction of the default mode network (DMN), the central executive network (CEN), and the salience network (SN) in Session 2.

## Data Availability

The data used in this study were taken from the open‐access data set (Gorgolewski et al., 2015). The data can be accessed using the following link https://openneuro.org/datasets/ds001168/versions/1.0.1. Analyzed data are available upon request from the corresponding author.
