# Supplementary material for: Test–retest stability of spontaneous brain activity and functional connectivity in the core resting‐state networks assessed with ultrahigh field 7‐Tesla resting‐state functional magnetic resonance imaging
Source: Hum Brain Mapp. 2022 Jan 19;43(6):2026–40. doi: 10.1002/hbm.25771 (PMC8933332; doi:10.1002/hbm.25771)
Supplement: Supplementary file 1 — FIGURE S1 Mean of the intermeasurement stability across 15 subjects (calculated from the sample excluding one potential outlier subject) for the fMRI parameters (amplitude of low‐frequency fluctuations [ALFF], fractional ALFF [fALFF], regional homogeneity [ReHo], and degree centrality [DC]) in each core network, that is, the default mode network (DMN), the central executive network (CEN), and the salience network (SN). The error bars represent SD. [file HBM-43-2026-s001.docx]

# Supplementary Material

**Supplementary Figure 1 (S-Fig. 1)**


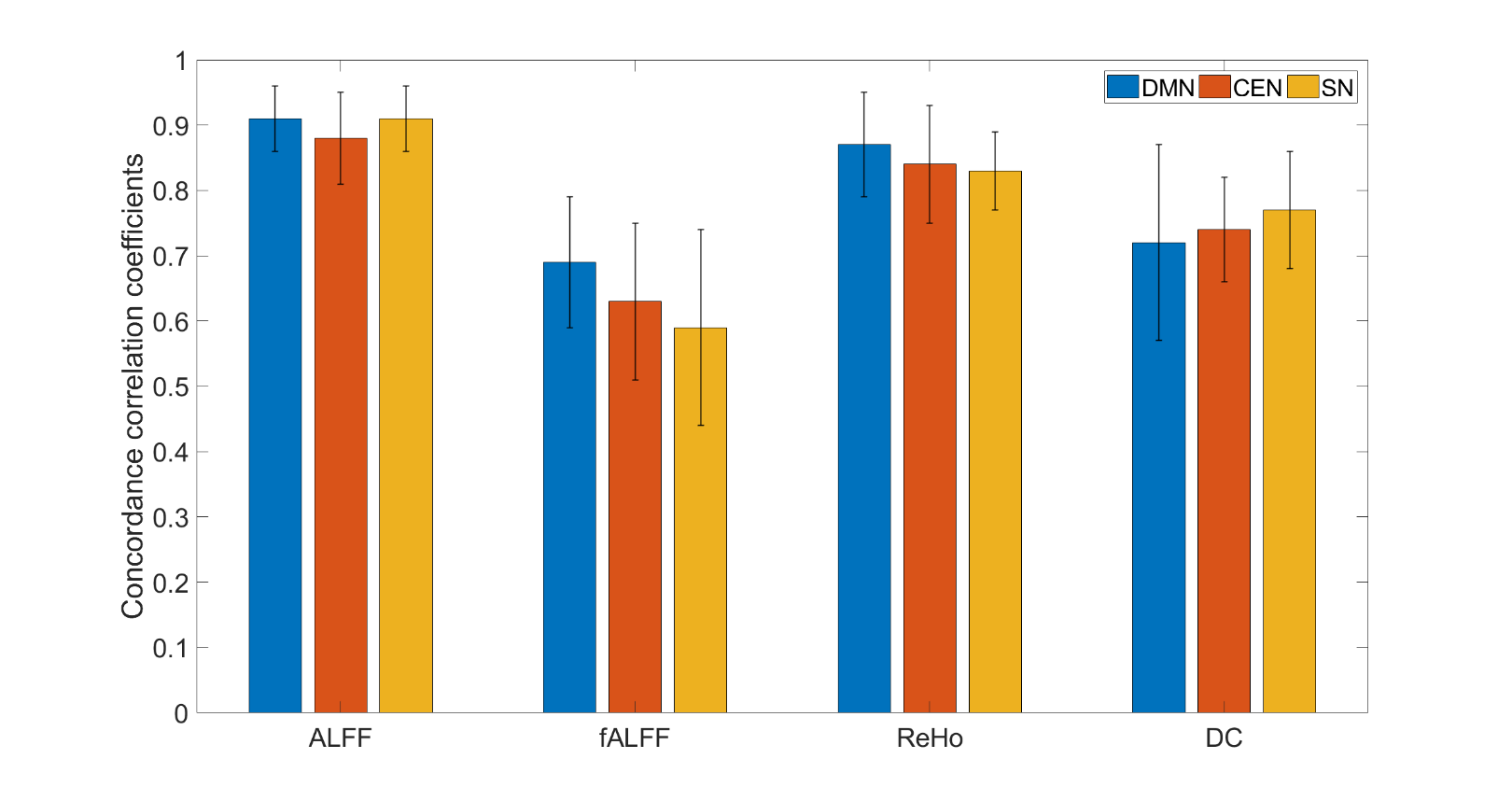


S-Fig. 1. Mean of the inter-measurement stability across 15 subjects (calculated from the sample excluding one potential outlier subject) for the fMRI parameters (amplitude of low-frequency fluctuations (ALFF), fractional ALFF (fALFF), regional homogeneity (ReHo), and degree centrality (DC)) in each core network, i.e., the default mode network (DMN), the central executive network (CEN), and the salience network (SN). The error bars represent standard deviation.

Generally, the results did not differ noticeably from the findings from the whole sample with 16 participants. We observed the mean stability of ALFF parameter to be strong in all three networks with highest in both, the DMN (0.91 ± 0.05; range from 0.76 to 0.96), and the SN (0.91 ± 0.05; range from 0.76 to 0.95) followed by the CEN (0.88 ± 0.07; range from 0.69 to 0.95).

The mean stability of fALFF parameter was found to be moderate in all three networks: DMN: 0.69 ± 0.10 (range from 0.44 to 0.85); CEN: 0.63 ± 0.12 (range from 0.44 to 0.90); SN: 0.59 ± 0.15 (range from 0.35 to 0.83).

The mean stability of ReHo parameter was found to be strong in all three networks: DMN: 0.87 ± 0.08 (range from 0.64 to 0.93); CEN: 0.84 ± 0.09 (range from 0.56 to 0.94); SN: 0.83 ± 0.06 (range from 0.69 to 0.91).

The mean stability of DC parameter was found to be strong in all three networks with highest in the SN: 0.77 ± 0.09 (range from 0.64 to 0.92) followed by the CEN: 0.74 ± 0.08 (range from 0.62 to 0.90); and the DMN: 0.72 ± 0.15 (range from 0.32 to 0.89).
