# Supplementary material for: Test–retest stability of spontaneous brain activity and functional connectivity in the core resting‐state networks assessed with ultrahigh field 7‐Tesla resting‐state functional magnetic resonance imaging
Source: Hum Brain Mapp. 2022 Jan 19;43(6):2026–40. doi: 10.1002/hbm.25771 (PMC8933332; doi:10.1002/hbm.25771)
Supplement: Supplementary file 2 — FIGURE S2 Mean of the internetwork correlations stability between the three core resting‐state networks depicted as the mean correlations between the DMN/SN, the DMN/CEN, and the SN/CEN. The error bars represent SD. This calculation was performed on the sample of 15 participants, that is, without the one participant who was considered as a potential outlier. [file HBM-43-2026-s005.docx]

# Supplementary Material

**Supplementary Figure 2 (S-Fig. 2)**


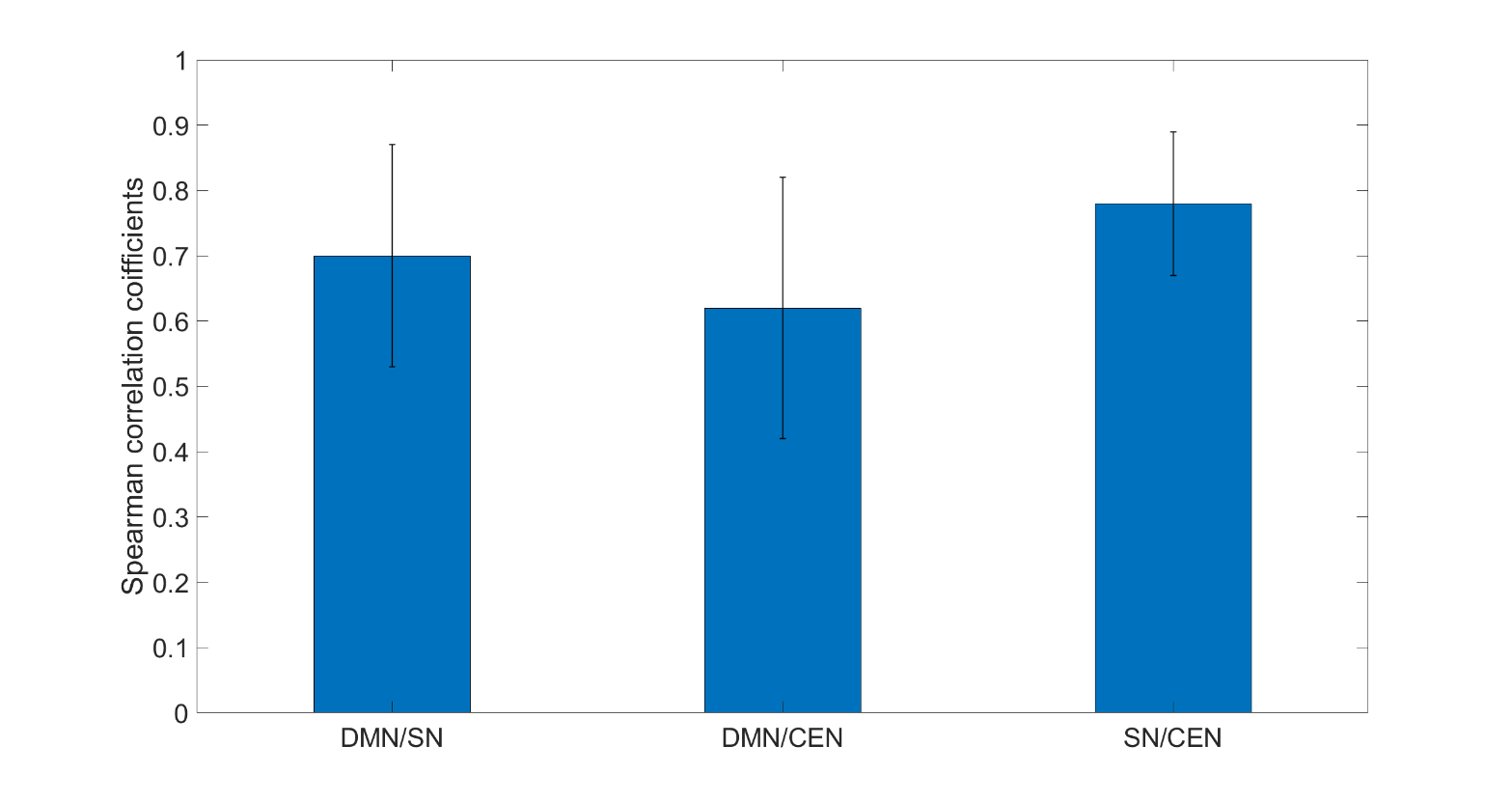


S-Fig.2. Mean of the inter-network correlations stability between the three core resting state networks depicted as the mean correlations between the DMN/SN, the DMN/CEN, and the SN/CEN. The error bars represent standard deviation. This calculation was performed on the sample of 15 participants, i.e., without the one participant who was considered as a potential outlier.

The inter-network connectivity showed a variant stability between the investigated network-pairs: between DMN and SN: 0.70 ± 0.17; (range from 0.36 to 0.94) strong stability; between DMN and CEN: 0.62 ± 0.21 (range from 0.12 to 0.94) moderate stability; between CEN and SN: 0.78 ± 0.11 (range from 0.60 to 0.91) strong stability.
