# Supplementary material for: Test–retest stability of spontaneous brain activity and functional connectivity in the core resting‐state networks assessed with ultrahigh field 7‐Tesla resting‐state functional magnetic resonance imaging
Source: Hum Brain Mapp. 2022 Jan 19;43(6):2026–40. doi: 10.1002/hbm.25771 (PMC8933332; doi:10.1002/hbm.25771)
Supplement: Supplementary file 3 — TABLE S1 Showing the included and excluded subjects in our study. Some subjects were excluded due to motion, missing of the data, or low‐resolution fMRI images. [file HBM-43-2026-s006.docx]

# Supplementary Material

**Supplementary Table 1 (S-Tab. 1)**

| **Subjects number in original data set** | **Excluding criteria** | **Subjects number in this study** |
| --- | --- | --- |
| 27430 | Included in study | Sub 01 |
| 27431 | Included in study | Sub 02 |
| 27432 | Included in study | Sub 03 |
| 27433 | Included in study | Sub 04 |
| 27434 | Included in study | Sub 05 |
| 27435 | Included in study | Sub 06 |
| 27436 | Included in study | Sub 07 |
| 27437 | Included in study | Sub 08 |
| 27438 | Excluded due to the motion (more than 2.0 mm in session 1) | *** |
| 27439 | Included to study | Sub 09 |
| 27440 | Excluded due to the low-resolution of fMRI images in both sessions | *** |
| 27441 | Included in study | Sub 10 |
| 27442 | Included in study | Sub 11 |
| 27443 | Included in study | Sub 12 |
| 27444 | Excluded due to fMRI data missing in the right frontal region | *** |
| 27445 | Excluded due to the motion (more than 2.5 mm in session 2) | *** |
| 27446 | Included in study | Sub 13 |
| 27447 | Included in study | Sub 14 |
| 27448 | Included in study | Sub 15 |
| 27449 | Included in study | Sub 16 |
| 27450 | Excluded due to motion (more than 3.0 mm in session 1) | *** |
| 27451 | Excluded due to the motion (more than 2.5 mm in session 2) | *** |

S-Tab.1. Showing the included and excluded subjects in our study. Some subjects were excluded due to motion, missing of the data, or low-resolution fMRI images.
