# Supplementary material for: Test–retest stability of spontaneous brain activity and functional connectivity in the core resting‐state networks assessed with ultrahigh field 7‐Tesla resting‐state functional magnetic resonance imaging
Source: Hum Brain Mapp. 2022 Jan 19;43(6):2026–40. doi: 10.1002/hbm.25771 (PMC8933332; doi:10.1002/hbm.25771)
Supplement: Supplementary file 4 — TABLE S2 Showing the percentage of volumes left for each subject and session after scrubbing. [file HBM-43-2026-s004.docx]

# Supplementary Material

**Supplementary Table 2 (S-Tab. 2)**

| **Subject number** | **Percentage volumes left in Session 1** | **Percentage volumes left in Session 2** |
| --- | --- | --- |
| **1** | 91 | 99 |
| **2** | 100 | 100 |
| **3** | 99 | 95 |
| **4** | 99 | 99 |
| **5** | 93 | 99 |
| **6** | 99 | 99 |
| **7** | 98 | 100 |
| **8** | 100 | 90 |
| **9** | 98 | 87 |
| **10** | 96 | 88 |
| **11** | 100 | 100 |
| **12** | 100 | 100 |
| **13** | 99 | 100 |
| **14** | 99 | 100 |
| **15** | 98 | 100 |
| **16** | 91 | 79 |

S-Tab.2. Showing the percentage of volumes left for each subject and session after scrubbing.
