# Supplementary material for: Test–retest stability of spontaneous brain activity and functional connectivity in the core resting‐state networks assessed with ultrahigh field 7‐Tesla resting‐state functional magnetic resonance imaging
Source: Hum Brain Mapp. 2022 Jan 19;43(6):2026–40. doi: 10.1002/hbm.25771 (PMC8933332; doi:10.1002/hbm.25771)
Supplement: Supplementary file 5 — TABLE S3 The concordance correlation coefficients and their 95 confidence interval level of significance for the fMRI parameters between the two sessions in the default mode network (DMN), the central executive network (CEN), and the salience network (SN). [file HBM-43-2026-s002.docx]

# Supplementary Material

**Supplementary Table 3 (S-Tab. 3)**

| **Subjects** |  | **DMN** | | |  | **CEN** | | |  | **SN** | | |
| --- | --- | --- | --- | --- | --- | --- | --- | --- | --- | --- | --- | --- |
|  | **ALFF** | **fALFF** | **ReHo** | **DC** | **ALFF** | **fALFF** | **ReHo** | **DC** | **ALFF** | **fALFF** | **ReHo** | **DC** |
| Sub01 | 0.72, CI [0.71, 0.74] | 0.19, CI [0.15, 0.22] | 0.77, CI [0.75, 0.78] | 0.4, CI [0.37, 0.42] | 0.75, CI [0.73, 0.77] | 0.02, CI [-0.03, 0.07] | 0.75, CI [0.73, 0.77] | 0.25, CI [0.21, 0.29] | 0.74, CI [0.72, 0.76] | (-0.20), CI [-0.24, -0.15] | 0.7, CI [0.68, 0.73] | 0.24, CI [0.21, 0.27] |
| Sub02 | 0.93, CI [0.92, 0.93] | 0.76, CI [0.74, 0.77] | 0.87, CI [0.86, 0.88] | 0.75, CI [0.73, 0.76] | 0.88, CI [0.87, 0.89] | 0.69, CI [0.67, 0.71] | 0.88, CI [0.87, 0.89] | 0.73, CI [0.71, 0.75] | 0.92, CI [0.92, 0.93] | 0.67, CI [0.65, 0.69] | 0.89, CI [0.89, 0.90] | 0.81, CI [0.79, 0.82] |
| Sub03 | 0.84, CI [0.83, 0.85] | 0.44, CI [0.42, 0.47] | 0.64, CI [0.62, 0.66] | 0.47, CI [0.44, 0.49] | 0.69, CI [0.67, 0.71] | 0.44, CI [0.40, 0.48] | 0.56, CI [0.53, 0.59] | 0.62, CI [0.59, 0.65] | 0.87, CI [0.85, 0.88] | 0.35, CI [0.31, 0.40] | 0.76, CI [0.74, 0.77] | 0.78, CI [0.76, 0.80] |
| Sub04 | 0.93, CI [0.92, 0.93] | 0.8, CI [0.79, 0.81] | 0.92, CI [0.92, 0.93] | 0.74, CI [0.72, 0.75] | 0.85, CI [0.84, 0.87] | 0.62, CI [0.59, 0.65] | 0.89, CI [0.88, 0.90] | 0.8, CI [0.78, 0.82] | 0.91, CI [0.91, 0.92] | 0.66, CI [0.64, 0.69] | 0.88, CI [0.87, 0.89] | 0.84, CI [0.83, 0.86] |
| Sub05 | 0.76, CI [0.75, 0.78] | 0.74, CI [0.73, 0.76] | 0.93, CI [0.93, 0.94] | 0.87, CI [0.86, 0.88] | 0.83, CI [0.81, 0.84] | 0.52, CI [0.48, 0.55] | 0.84, CI [0.82, 0.85] | 0.8, CI [0.78, 0.82] | 0.76, CI [0.74, 0.78] | 0.41, CI [0.37, 0.45] | 0.84, CI [0.83, 0.85] | 0.81, CI [0.79, 0.82] |
| Sub06 | 0.96, CI [0.95, 0.96] | 0.7, CI [0.68, 0.72] | 0.9, CI [0.89, 0.91] | 0.61, CI [0.59, 0.63] | 0.9, CI [0.89, 0.91] | 0.51, CI [0.47, 0.54] | 0.84, CI [0.82, 0.85] | 0.65, CI [0.61, 0.68] | 0.93, CI [0.93, 0.94] | 0.37, CI [0.34, 0.41] | 0.85, CI [0.84, 0.86] | 0.67, CI [0.64, 0.69] |
| Sub07 | 0.9, CI [0.89, 0.90] | 0.61, CI [0,60 0.63] | 0.84, CI [0,83 0.85] | 0.76, CI [0.75, 0.78] | 0.85, CI [0.84, 0.87] | 0.56, CI [0.52, 0.59] | 0.82, CI [0.80, 0.83] | 0.77, CI [0.75, 0.79] | 0.9, CI [0.89, 0.91] | 0.41, CI [0.37, 0.44] | 0.79, CI [0.77, 0.80] | 0.64, CI [0.61, 0.66] |
| Sub08 | 0.93, CI [0.93, 0.93] | 0.62, CI [0.60, 0.64] | 0.81, CI [0.80, 0.82] | 0.71, CI [0.69, 0.72] | 0.9, CI [0.89, 0.91] | 0.5, CI [0.46, 0.53] | 0.73, CI [0.71, 0.76] | 0.7, CI [0.68, 0.73] | 0.95, CI [0.95, 0.95] | 0.46, CI [00.42, 0.49] | 0.79, CI [0.77, 0.81] | 0.64, CI [0.62, 0.66] |
| Sub09 | 0.96, CI [0.95, 0.96] | 0.85, CI [0.84, 0.86] | 0.91, CI [0.90, 0.91] | 0.89, CI [0.88, 0.90] | 0.94, CI [0.93, 0.94] | 0.9, CI [0.89, 0.91] | 0.94, CI [0.93, 0.95] | 0.9, CI [0.88, 0.90] | 0.92, CI [0.92, 0.93] | 0.75, CI [0.73, 0.77] | 0.84, CI [0.83, 0.86] | 0.9, CI [0.89, 0.91] |
| Sub10 | 0.93, CI [0.92, 0.93] | 0.75, CI [0.74, 0.76] | 0.92, CI [0.91, 0.92] | 0.83, CI [0.82, 0.84] | 0.92, CI [0.91, 0.93] | 0.63, CI [0.60, 0.65] | 0.9, CI [0.89, 0.91] | 0.71, CI [0.69, 0.73] | 0.95, CI [0.95, 0.96] | 0.58, CI [0.55, 0.61] | 0.81, CI [0.79, 0.82] | 0.67, CI [0.65, 0.69] |
| Sub11 | 0.95, CI [0.95, 0.95] | 0.75, CI [0.73, 0.76] | 0.91, CI [0.91, 0.92] | 0.76, CI [0.74, 0.77] | 0.95, CI [0.95, 0.96] | 0.76, CI [0.74, 0.79] | 0.93, CI [0.92, 0.94] | 0.77, CI [0.75, 0.80] | 0.92, CI [0.91, 0.92] | 0.69, CI [0.66, 0.71] | 0.86, CI [0.85, 0.87] | 0.78, CI [0.76, 0.80] |
| Sub12 | 0.89, CI [0.89, 0.90] | 0.59, CI [.58, 0.61] | 0.89, CI [0.88, 0.89] | 0.75, CI [0.73, 0.76] | 0.87, CI [0.86, 0.88] | 0.61, CI [0.58, 0.63] | 0.91, CI [0.90, 0.92] | 0.67, CI [0.65, 0.70] | 0.9, CI [0.89, 0.91] | 0.59, CI [0.56, 0.61] | 0.87, CI [0.86, 0.88] | 0.78, CI [0.76, 0.79] |
| Sub13 | 0.93, CI [0.93, 0.93] | 0.73, CI [0.72, 0.75] | 0.92, CI [0.92, 0.93] | 0.71, CI [0.69, 0.73] | 0.93, CI [0.92, 0.93] | 0.69, CI [0.66, 0.72] | 0.87, CI [0.86, 0.89] | 0.71, CI [0.69, 0.74] | 0.89, CI [0.88, 0.90] | 0.73, CI [0.71, 0.75] | 0.83, CI [0.82, 0.85] | 0.66, CI [0.64, 0.69] |
| Sub14 | 0.91, CI [0.91, 0.92] | 0.72, CI [0.71, 0.74] | 0.91, CI [0.90, 0.91] | 0.72, CI [0.70, 0.73] | 0.81, CI [0.80, 0.83] | 0.72, CI [0.70, 0.75] | 0.8, CI [0.78, 0.82] | 0.73, CI [0.70, 0.75] | 0.9, CI [0.89, 0.91] | 0.75, CI [0.73, 0.76] | 0.69, CI [0.67, 0.72] | 0.87, CI [0.85, 0.88] |
| Sub15 | 0.91, CI [0.91, 0.92] | 0.60, CI [0.59, 0.62] | 0.74, CI [0.73, 0.75] | 0.32, CI [0.31, 0.34] | 0.92, CI [0.91, 0.93] | 0.73, CI [0.71, 0.75] | 0.83, CI [0.82, 0.85] | 0.63, CI [0.61, 0.65] | 0.95, CI [0.95, 0.96] | 0.83, CI [0.82, 0.84] | 0.91, CI [0.90, 0.91] | 0.72, CI [0.70, 0.74] |
| Sub16 | 0.95, CI [0.95, 0.96] | 0.68, CI [0.66, 0.70] | 0.93, CI [0.92, 0.93] | 0.88, CI [0.87, 0.89] | 0.91, CI [0.90, 0.92] | 0.64, CI [0.61, 0.67] | 0.89, CI [0.88, 0.90] | 0.88, CI [0.87, 0.89] | 0.95, CI [0.94, 0.95] | 0.61, CI [0.58, 0.64] | 0.91, CI [0.90, 0.92] | 0.92, CI [0.91, 0.92] |

S-Tab.3. The concordance correlation coefficients and their 95 confidence interval level of significance for the fMRI parameters between the two sessions in the default mode network (DMN), the central executive network (CEN), and the salience network (SN).
