# Supplementary material for: Test–retest stability of spontaneous brain activity and functional connectivity in the core resting‐state networks assessed with ultrahigh field 7‐Tesla resting‐state functional magnetic resonance imaging
Source: Hum Brain Mapp. 2022 Jan 19;43(6):2026–40. doi: 10.1002/hbm.25771 (PMC8933332; doi:10.1002/hbm.25771)
Supplement: Supplementary file 6 — TABLE S4 The average values of the inter‐network interaction of the default mode network (DMN), the central executive network (CEN), and the salience network (SN) in Session 1. [file HBM-43-2026-s003.docx]

# Supplementary Material

**Supplementary Table 4 (S-Tab. 4)**

| **Networks** |  | DMN | | | | SN | | | | | | | CEN | | | |
| --- | --- | --- | --- | --- | --- | --- | --- | --- | --- | --- | --- | --- | --- | --- | --- | --- |
|  | **Sub-regions** | **MPFC** | **LP (L)** | **LP (R)** | **PCC** | **ACC** | **AInsula (L)** | **AInsula (R)** | **RPFC (L)** | **RPFC (R)** | **SMG (L)** | **SMG (R)** | **LPFC (L)** | **PPC (L)** | **LPFC (R)** | **PPC (R)** |
| DMN | **MPFC** | Inf | 0.51 | 0.57 | 0.65 | 0.37 | 0.18 | 0.11 | 0.28 | 0.23 | 0.07 | 0.02 | 0.30 | 0.30 | 0.31 | 0.28 |
|  | **LP (L)** | 0.51 | Inf | 0.89 | 0.71 | 0.14 | 0.05 | -0.03 | 0.21 | 0.13 | 0.14 | 0.12 | 0.42 | 0.40 | 0.27 | 0.25 |
|  | **LP (R)** | 0.57 | 0.89 | Inf | 0.73 | 0.21 | 0.15 | 0.08 | 0.25 | 0.21 | 0.25 | 0.24 | 0.35 | 0.30 | 0.41 | 0.39 |
|  | **PCC** | 0.65 | 0.71 | 0.73 | Inf | 0.45 | 0.28 | 0.20 | 0.48 | 0.42 | 0.23 | 0.23 | 0.42 | 0.34 | 0.47 | 0.39 |
| SN | **ACC** | 0.37 | 0.14 | 0.21 | 0.45 | Inf | 0.89 | 0.82 | 0.87 | 0.79 | 0.54 | 0.50 | 0.35 | 0.22 | 0.41 | 0.28 |
|  | **AInsula (L)** | 0.18 | 0.05 | 0.15 | 0.28 | 0.89 | Inf | 0.95 | 0.81 | 0.61 | 0.72 | 0.60 | 0.38 | 0.14 | 0.34 | 0.22 |
|  | **AInsula (R)** | 0.11 | -0.03 | 0.08 | 0.20 | 0.82 | 0.95 | Inf | 0.66 | 0.73 | 0.67 | 0.73 | 0.19 | 0.08 | 0.35 | 0.27 |
|  | **RPFC (L)** | 0.28 | 0.21 | 0.25 | 0.48 | 0.87 | 0.81 | 0.66 | Inf | 1.02 | 0.62 | 0.52 | 0.49 | 0.30 | 0.45 | 0.36 |
|  | **RPFC (R)** | 0.23 | 0.13 | 0.21 | 0.42 | 0.79 | 0.61 | 0.73 | 1.02 | Inf | 0.52 | 0.62 | 0.29 | 0.15 | 0.56 | 0.43 |
|  | **SMG (L)** | 0.07 | 0.14 | 0.25 | 0.23 | 0.54 | 0.72 | 0.67 | 0.62 | 0.52 | Inf | 0.86 | 0.20 | 0.09 | 0.22 | 0.21 |
|  | **SMG (R)** | 0.02 | 0.12 | 0.24 | 0.23 | 0.50 | 0.60 | 0.73 | 0.52 | 0.62 | 0.86 | Inf | 0.09 | 0.01 | 0.22 | 0.22 |
| CEN | **LPFC (L)** | 0.30 | 0.42 | 0.35 | 0.42 | 0.35 | 0.38 | 0.19 | 0.49 | 0.29 | 0.20 | 0.09 | Inf | 0.88 | 0.84 | 0.56 |
|  | **PPC (L)** | 0.30 | 0.40 | 0.30 | 0.34 | 0.22 | 0.14 | 0.08 | 0.30 | 0.15 | 0.09 | 0.01 | 0.88 | Inf | 0.53 | 0.79 |
|  | **LPFC (R)** | 0.31 | 0.27 | 0.41 | 0.47 | 0.41 | 0.34 | 0.35 | 0.45 | 0.56 | 0.22 | 0.22 | 0.84 | 0.53 | Inf | 0.94 |
|  | **PPC (R)** | 0.28 | 0.25 | 0.39 | 0.39 | 0.28 | 0.22 | 0.27 | 0.36 | 0.43 | 0.21 | 0.22 | 0.56 | 0.79 | 0.94 | Inf |

S-Tab.4. The average values of the inter-network interaction of the default mode network (DMN), the central executive network (CEN), and the salience network (SN) in session 1.
