# Supplementary material for: Test–retest stability of spontaneous brain activity and functional connectivity in the core resting‐state networks assessed with ultrahigh field 7‐Tesla resting‐state functional magnetic resonance imaging
Source: Hum Brain Mapp. 2022 Jan 19;43(6):2026–40. doi: 10.1002/hbm.25771 (PMC8933332; doi:10.1002/hbm.25771)
Supplement: Supplementary file 7 — TABLE S5 The average values of the internetwork interaction of the default mode network (DMN), the central executive network (CEN), and the salience network (SN) in Session 2. [file HBM-43-2026-s007.docx]

# Supplementary Material

**Supplementary Table 5 (S-Tab. 5)**

| **Networks** |  | DMN | | | | SN | | | | | | | CEN | | | |
| --- | --- | --- | --- | --- | --- | --- | --- | --- | --- | --- | --- | --- | --- | --- | --- | --- |
|  | **Sub-regions** | **MPFC** | **LP (L)** | **LP (R)** | **PCC** | **ACC** | **AInsula (L)** | **AInsula (R)** | **RPFC (L)** | **RPFC (R)** | **SMG (L)** | **SMG (R)** | **LPFC (L)** | **PPC (L)** | **LPFC (R)** | **PPC (R)** |
| DMN | **MPFC** | Inf | 0.55 | 0.60 | 0.68 | 0.38 | 0.11 | 0.12 | 0.32 | 0.27 | 0.03 | 0.03 | 0.30 | 0.35 | 0.37 | 0.38 |
|  | **LP (L)** | 0.55 | Inf | 0.94 | 0.68 | 0.18 | 0.00 | -0.01 | 0.25 | 0.18 | 0.15 | 0.14 | 0.35 | 0.40 | 0.31 | 0.32 |
|  | **LP (R)** | 0.60 | 0.94 | Inf | 0.75 | 0.21 | 0.09 | 0.05 | 0.27 | 0.25 | 0.18 | 0.20 | 0.32 | 0.31 | 0.47 | 0.50 |
|  | **PCC** | 0.68 | 0.68 | 0.75 | Inf | 0.45 | 0.20 | 0.21 | 0.48 | 0.47 | 0.14 | 0.21 | 0.34 | 0.31 | 0.50 | 0.44 |
| SN | **ACC** | 0.38 | 0.18 | 0.21 | 0.45 | Inf | 0.81 | 0.72 | 0.90 | 0.79 | 0.46 | 0.45 | 0.36 | 0.21 | 0.38 | 0.24 |
|  | **AInsula (L)** | 0.11 | 0.00 | 0.09 | 0.20 | 0.81 | Inf | 0.94 | 0.72 | 0.60 | 0.66 | 0.55 | 0.31 | 0.09 | 0.23 | 0.13 |
|  | **AInsula (R)** | 0.12 | -0.01 | 0.05 | 0.21 | 0.72 | 0.94 | Inf | 0.62 | 0.72 | 0.55 | 0.63 | 0.19 | 0.07 | 0.28 | 0.21 |
|  | **RPFC (L)** | 0.32 | 0.25 | 0.27 | 0.48 | 0.90 | 0.72 | 0.62 | Inf | 1.05 | 0.55 | 0.49 | 0.45 | 0.31 | 0.41 | 0.32 |
|  | **RPFC (R)** | 0.27 | 0.18 | 0.25 | 0.47 | 0.79 | 0.60 | 0.72 | 1.05 | Inf | 0.45 | 0.61 | 0.26 | 0.17 | 0.50 | 0.43 |
|  | **SMG (L)** | 0.03 | 0.15 | 0.18 | 0.14 | 0.46 | 0.66 | 0.55 | 0.55 | 0.45 | Inf | 0.79 | 0.16 | 0.09 | 0.13 | 0.11 |
|  | **SMG (R)** | 0.03 | 0.14 | 0.20 | 0.21 | 0.45 | 0.55 | 0.63 | 0.49 | 0.61 | 0.79 | Inf | 0.04 | -0.01 | 0.14 | 0.14 |
| CEN | **LPFC (L)** | 0.30 | 0.35 | 0.32 | 0.34 | 0.36 | 0.31 | 0.19 | 0.45 | 0.26 | 0.16 | 0.04 | Inf | 0.83 | 0.77 | 0.45 |
|  | **PPC (L)** | 0.35 | 0.40 | 0.31 | 0.31 | 0.21 | 0.09 | 0.07 | 0.31 | 0.17 | 0.09 | -0.01 | 0.83 | Inf | 0.56 | 0.79 |
|  | **LPFC (R)** | 0.37 | 0.31 | 0.47 | 0.50 | 0.38 | 0.23 | 0.28 | 0.41 | 0.50 | 0.13 | 0.14 | 0.77 | 0.56 | Inf | 0.93 |
|  | **PPC (R)** | 0.38 | 0.32 | 0.50 | 0.44 | 0.24 | 0.13 | 0.21 | 0.32 | 0.43 | 0.11 | 0.14 | 0.45 | 0.79 | 0.93 | Inf |

S-Tab.5. The average values of the inter-network interaction of the default mode network (DMN), the central executive network (CEN), and the salience network (SN) in session 2.
